# Auto-antibodies neutralizing type I interferons in ~10% of Moroccan patients with life-threatening COVID-19

**DOI:** 10.3389/fimmu.2026.1870598

**Published:** 2026-07-14

**Authors:** Halima Kholaiq, Adrian Gervais, Paul Bastard, Hind Ouair, Lucy Bizien, Amyrath Géraldo, Fatima Ailal, Ibtihal Benhsaien, Assiya El Kettani, Ouissal Aissaoui, Naima Amenzoui, Chafik El Kettani El Hamidi, Boubaker Charra, Maha Soussi Abdallaoui, Rachid Al Harrar, Lahoucine Barrou, Mohamed Benghanem Gharbi, Moulay Hicham Afif, Qian Zhang, Laurent Abel, Aurélie Cobat, Anne Puel, Emmanuelle Jouanguy, Jean-Laurent Casanova, Ahmed Aziz Bousfiha, Jalila El Bakkouri

**Affiliations:** 1 Laboratory of Clinical Immunology, Infection And Autoimmunity (LICIA), Faculty of Medicine and Pharmacy, Hassan II University of Casablanca, Casablanca, Morocco; 2 Laboratory of Human Genetics of Infectious Diseases, Institut National de la Sante et de la Recherche Medicale (INSERM) U1163, Necker Hospital for Sick Children, Paris, France; 3 Imagine Institute, Paris Cité University, Paris, France; 4 St. Giles Laboratory of Human Genetics of Infectious Diseases, The Rockefeller University, New York, NY, United States; 5 Pediatric Hematology-Immunology and Rheumatology Unit, Necker Hospital for Sick Children, Assistance Publique-Hôpitaux de Paris (AP-HP), Paris, France; 6 Department of Clinical Immunology and Infectious Pediatrics, Abderrahim Harouchi Hospital, Ibn Rochd University Hospital Center, Casablanca, Morocco; 7 Laboratory of Bacteriology, Virology and Hospital Hygiene, Ibn Rochd University Hospital Centre, Casablanca, Morocco; 8 Laboratory of Bacteriology and Virology, Faculty of Medicine and Pharmacy, Hassan II University of Casablanca, Casablanca, Morocco; 9 Pediatric Anesthesiology and Intensive Care Unit, Ibn Rochd University Hospital, Casablanca, Morocco; 10 Surgical Intensive Care and Anesthesia Department, Ibn Rochd University Hospital of Casablanca, Faculty of Medicine and Pharmacy-Hassan II University of Casablanca, Casablanca, Morocco; 11 Anesthesia and Critical Care, Cheikh Khalifa International University Hospital, Mohammed VI University of Science and Health, Casablanca, Morocco; 12 Department of Intensive Care and Anesthesiology, Ibn Rochd University Hospital Center, Casablanca, Morocco; 13 Laboratory of Parasitology-Mycology, Ibn Rochd University Hospital Center, Casablanca, Morocco; 14 Anesthesiology and Critical Care, Ibn Rochd University Hospital Center, Casablanca, Morocco; 15 Surgical Emergencies Intensive Care and Anesthesia Department, Ibn Rochd University Hospital Center, Casablanca, Morocco; 16 Nephrology, Dialysis and Renal Transplantation Unit, Ibn Rochd University Hospital Center, Casablanca, Morocco; 17 Pneumology Department, 20 Août Hospital, Ibn Rochd University Hospital Center, Casablanca, Morocco; 18 Howard Hughes Medical Institute, New York, NY, United States; 19 Department of Pediatrics, Necker Hospital for Sick Children, Assistance Publique-HÔpitaux de Paris (AP-HP), Paris, France; 20 Laboratory of Immunology, Ibn Rochd University Hospital Center of Casablanca, Casablanca, Morocco; 21 Immunopathology-Immunotherapy-Immunomonitoring Laboratory, Faculty of Medicine, Mohammed VI University of Science and Health, Casablanca, Morocco

**Keywords:** autoantibodies, COVID-19, inborn errors of immunity, SARS-CoV-2, type-I interferons, viral infection

## Abstract

**Introduction:**

Autoantibodies neutralizing type I interferon (AAN-I-IFN) have been found in at least 10-15% of critical COVID-19 pneumonia cases in various studies across North and Latin America, Oceania, Europe, and Asia. We sought to analyze the prevalence of AAN-I-IFN and describe the demographic, clinical, and laboratory characteristics in a Moroccan cohort of patients with life-threatening COVID-19.

**Methods:**

We performed a cross-sectional and multicenter study of patients hospitalized in different university hospitals and clinical centers in Casablanca between November 2020 and December 2021.

**Results:**

Our cohort included 195 patients with proven SARS-CoV-2 infection, 164 (84.1%) of whom developed severe or critical COVID-19 disease requiring hospitalization, and 31 (15.9%) patients developed mild or moderate disease. Patients with moderate or mild COVID-19 did not exhibit detectable levels of AAN-I-IFNs. Among the 20 patients with AAN-I-IFN, most were men (n=16, 80%), and age ranged from 19 to 101 years. 13 (65%) patients with severe COVID-19 and 7 (35%) with critical COVID-19 had AAN-I-IFNs. Twelve patients (60%) had autoantibodies (auto-Abs) neutralizing both high and low concentrations of IFN-α2 and/or IFN-ω, five (25%) had auto-Abs neutralizing only low concentrations of IFN-α2 or IFN-ω, two (10%) had auto-Abs neutralizing IFN-β (1ng/mL) only, and one (5%) had auto-Abs neutralizing high and low concentrations of IFN-α2 and IFN-ω, as well as IFN-β at 1ng/mL, but none neutralized IFN-β at 10 ng/mL.

**Conclusion:**

Overall, AAN-I-IFNs were detected in 20/195 (10.3%) of Moroccan patients with life-threatening COVID-19 and in 20/164 (12.2%) patients with severe or critical disease, whereas none were detected in patients with mild or moderate COVID-19.

## 1 Introduction

Since late 2019, the novel coronavirus Severe Acute Respiratory Syndrome Coronavirus 2 (SARS-CoV-2), the causative agent of coronavirus disease 2019 (COVID-19), has spread worldwide, resulting in the most severe pandemic since the 1918 influenza pandemic. As of May 10, 2026, the World Health Organization (WHO) has reported more than 779 million confirmed cases of COVID-19 and over seven million deaths globally (1), including more than 1.3 million cases and 16.305 deaths in Morocco (1). Human respiratory viruses primarily affect the respiratory tract and can cause a wide range of clinical manifestations, ranging from mild upper respiratory infections to fatal respiratory disorders with multiorgan failure. One of the most striking features of SARS-CoV-2 infection is the marked inter-individual clinical variability (2, 3). In early pre-vaccine cohorts, approximately 15% of SARS-CoV-2-infected individuals developed severe COVID-19 requiring oxygen therapy, and about 5% developed critical disease requiring ventilatory support (4).

Type I interferons (IFNs), including the 12 interferon-α (IFN-α) subtypes, interferon-β (IFN-β), interferon-ϵ (IFN-ϵ), interferon-κ (IFN-κ), and interferon-ω (IFN-ω), are potent cytokines that play a crucial role in the innate immune defense against viral diseases (5). In the context of COVID-19, type I IFNs are essential for early antiviral protection by limiting viral replication and initiating the antiviral immune response (5, 6).

Anti-cytokine-neutralizing autoantibodies (auto-Abs) have been reported in various primary immunodeficiencies with autoimmune features, most commonly against type I IFNs, IL-17s, and IL-22, which impair the activity of their target cytokines, thereby compromising immune responses and increasing susceptibility to specific infectious diseases (7–9). AAN-I-IFN have been known since the early 1980s (10), but they were considered clinically silent until the COVID-19 pandemic (11). However, in 1981, AAN-I-IFN was reported in a 77-year-old otherwise healthy woman who developed a disseminated herpes zoster episode (12). They have since been reported in a growing number of life-threatening viral diseases, including critical influenza pneumonia in ~5% of cases (13), critical Middle East Respiratory Syndrome (MERS) pneumonia in approximately 20% of cases (14), and critical COVID-19 pneumonia in approximately 10-20% of cases (11) (as validated in multiple subsequent studies (15–51)). AAN-I-IFN have also been implicated in life-threatening adverse reactions to live-attenuated viral vaccines, including yellow fever virus vaccine strain 17D in approximately 30% of cases (52) and, more recently, chikungunya live-attenuated vaccine encephalitis (53). In addition, they have been detected in approximately 40% of patients with West Nile virus (WNV) encephalitis (54), ~10% of patients with severe tick-borne encephalitis (TBE) (55), and in rare severe arboviral diseases caused by Powassan virus (POWV), Usutu virus (USUV), and Ross River virus (RRV) (56). More recently, AAN-I-IFN have also been identified in patients with HSV-triggered fulminant viral hepatitis (FVH) (57). These auto-Abs may remain clinically silent in apparently healthy individuals until infection occurs, when they neutralize the early type I IFN response and predispose to viral disease (7, 11). Population studies have shown that neutralizing AAN-I-IFN are present in ~0.4% of apparently healthy individuals under 70 years of age, with a marked increase after 70 years, reaching ~4% (52).

Although the role of AAN-I-IFNs in the severity of COVID-19 has been robustly documented by several research groups in multiple cohorts worldwide, it remains uncharacterized in African populations. To address this knowledge gap, we studied a multicenter cohort of Moroccan patients with life-threatening COVID-19. We aimed to determine the prevalence of AAN-I-IFN in this cohort, compare disease severity among hospitalized patients with and without AAN-I-IFN, and to identify the clinical and biological characteristics associated with their presence.

## 2 Methods

### 2.1 Study design and participants

Written informed consent was obtained from all patients or their legal guardians. All patients were recruited according to protocols approved by the local research ethics committee of Ibn Rochd University Hospital. The present cross-sectional study included patients of all ages hospitalized with a confirmed SARS-CoV-2 infection, and those with or without comorbidities. Patients with human immunodeficiency virus were excluded. We recruited 195 patients between November 2, 2020, and December 1, 2021, from six clinical centers in Casablanca. 101 (51.8%) patients had confirmed critical COVID-19, and 63 (32.3%) patients had severe COVID-19. Additionally, 31/195 (15.9%) patients recruited had clinically confirmed mild (n=15, 7.7%) or moderate (n=16, 8.2%) SARS-CoV-2 infection. Of note, the minority of cases classified as moderate or mild (n = 31, 15.9%) were hospitalized due to risk factors such as advanced age or serious comorbidities requiring monitoring; however, they did not meet the criteria for severe or critical classification. Comprehensive clinical assessments and examinations were performed for all patients. Plasma samples were collected from all individuals for immunoassay testing to detect AAN-I-IFNs.

### 2.2 Nucleic acid extraction and reverse transcription-quantitative polymerase chain reaction

#### 2.2.1 Specimen collection

The detection of SARS-CoV-2 nucleic acids in patients’ swabs was performed in a total of 195 individuals with symptoms of SARS-CoV-2 infection. Nasopharyngeal or oropharyngeal swabs were collected from patients and transferred to the Laboratory of Bacteriology, Virology and Hospital Hygiene, Ibn Rochd University Hospital Centre, for qualitative *in vitro* detection of SARS-CoV-2 nucleic acids.

#### 2.2.2 RT-qPCR amplification

Detection of SARS-CoV-2 was performed using RT-qPCR assay, specifically the Moroccan Foundation for Advanced Science Innovation and Research (MAScIR) SARS-CoV-2 M 2.0 Kit and GeneFinder™ COVID-19 Plus RealAmp Kit.

#### 2.2.3 MAScIR SARS-CoV-2 M 2.0 Kit

The MAScIR SARS-CoV-2 M Kit is a qualitative multiplex amplification test, that enables the detection of SARS-CoV-2 in human specimens. It targets the two viral genes of SARS-CoV-2 (RNA-dependent RNA polymerase (RdRp) and Spike (S)) and an internal control of human cells, which is based on a one-step RT-qPCR. The same reaction was applied to all three targets. 2.5 μL of the enzyme mix (enzyme cocktail, deoxynucleotide triphosphates (dNTP), and reaction buffer), 1 μL of the primer and probe mix, and 6.5 μL of viral eluate containing purified SARS-CoV-2 RNA are all included in each reaction mixture. For every set of samples, three controls were used: positive, negative, and negative extraction. QuantStudio 7 Applied Biosystem thermal cycler was used for the amplification. The amplification program was detailed as follows: A reverse transcription step at 50 °C for five minutes, an activation step at 95 °C for twenty seconds, and a series of forty cycles of denaturation-hybridization-elongation (denaturation at 95 °C for three seconds and hybridization and elongation at 60 °C for thirty seconds). The total duration of the polymerase chain reaction (PCR) on the QuantStudio 7 Applied Biosystem was 56 minutes. A positive result was defined as a cycle threshold (Ct) value <30, and low positive if one or both targets (RdRp and S) had a Ct value between 31 and 36, Ct value of > 37 were considered negative.

#### 2.2.4 GeneFinder™ COVID-19 Plus RealAmp Kit

The GeneFinder™ kit enables qualitative detection of SARS-CoV-2 nucleic acids and internal cellular control and the detection of three viral targets (RdRp, Nucleocapsid (N), and Envelope (E)). 15 μL of the master mix and 5 μL of the patient sample eluate were combined in each reaction well. In every set of samples, both positive and negative controls were employed. The amplification, carried out on the QuantStudio 7 Applied Biosystem thermal cyclers, consists of the following steps: a reverse transcription step at 50 °C for 20 minutes, an activation step at 95 °C for 5 minutes, and a series of 45 denaturation-hybridization-elongation cycles (denaturing at 95 °C for 15 seconds and hybridization and elongation at 60 °C for 60 seconds). The duration of the PCR on the thermal cycler was 120 minutes).

### 2.3 COVID-19 classification

COVID-19 severity was assessed based on the Diagnosis and Treatment Protocol for Novel Coronavirus Pneumonia and according to established clinical criteria. Patients with “critical COVID-19” were defined as those who developed pneumonia requiring high-flow oxygen via high-concentration mask or mechanical ventilation. Pneumonia in patients requiring low-flow oxygen (<6 L/min) delivered via nasal cannula with peripheral capillary oxygen saturation (SpO_2_) <90% was classified as “severe COVID-19”. Patients with ‘‘Moderate COVID-19’’ were hospitalized with standard breathing support; and ‘‘Mild COVID-19’’ were not hospitalized, mild symptoms.

### 2.4 Laboratory variables

The Elecsys IL-6^©^ kit from Roche Diagnostics was used to perform the interleukin-6 (IL-6) assay. A non-competitive chemiluminescent immunoassay was used, in which 18 µL of sample is first treated with IL-6-specific antibodies and then with IL-6-specific antibodies labelled with ruthenium complexes. These complexes are magnetically trapped, and a chemiluminescent signal directly proportional to the IL-6 concentration is induced by voltage application. The IL-6 assay is designed for use with Cobas immunoassay analyzer. Lymphocyte, platelet, and neutrophil counts were measured from EDTA-anticoagulated peripheral blood samples using a Sysmex XS-550 analyzer. Serum ferritin levels were assessed using ARCHITECT ferritin assay (Abbott Diagnostics, USA). Serum C-Reactive Protein (CRP) was measured by immunoturbidimetry (CRP latex HS Roche kit, Roche Diagnostics). Patient samples were collected at the time of admission, centrifuged at 1,500 rpm for 15 min to obtain serum or plasma and treated promptly in the laboratory according to the manufacturer’s instructions. The samples were stored at −80 °C.

### 2.5 Luciferase reporter assays

The neutralizing activity of anti-IFN-α2, anti-IFN-ω, and anti-IFN-β autoantibodies was assessed using a reporter luciferase assay, as described previously by Bastard et al. (52). Briefly, human embryonic kidney (HEK293T) cells were transfected with a plasmid containing the firefly luciferase gene under control of the human interferon-stimulated response element (ISRE) promoter in the pGL4.45 backbone and a plasmid constitutively expressing Renilla luciferase for normalization (pRL-SV40). Cells were transfected using X-tremeGENE9 transfection reagent (Sigma-Aldrich, cat. no. 6365779001) for 24 hours.

**Figure 1:**
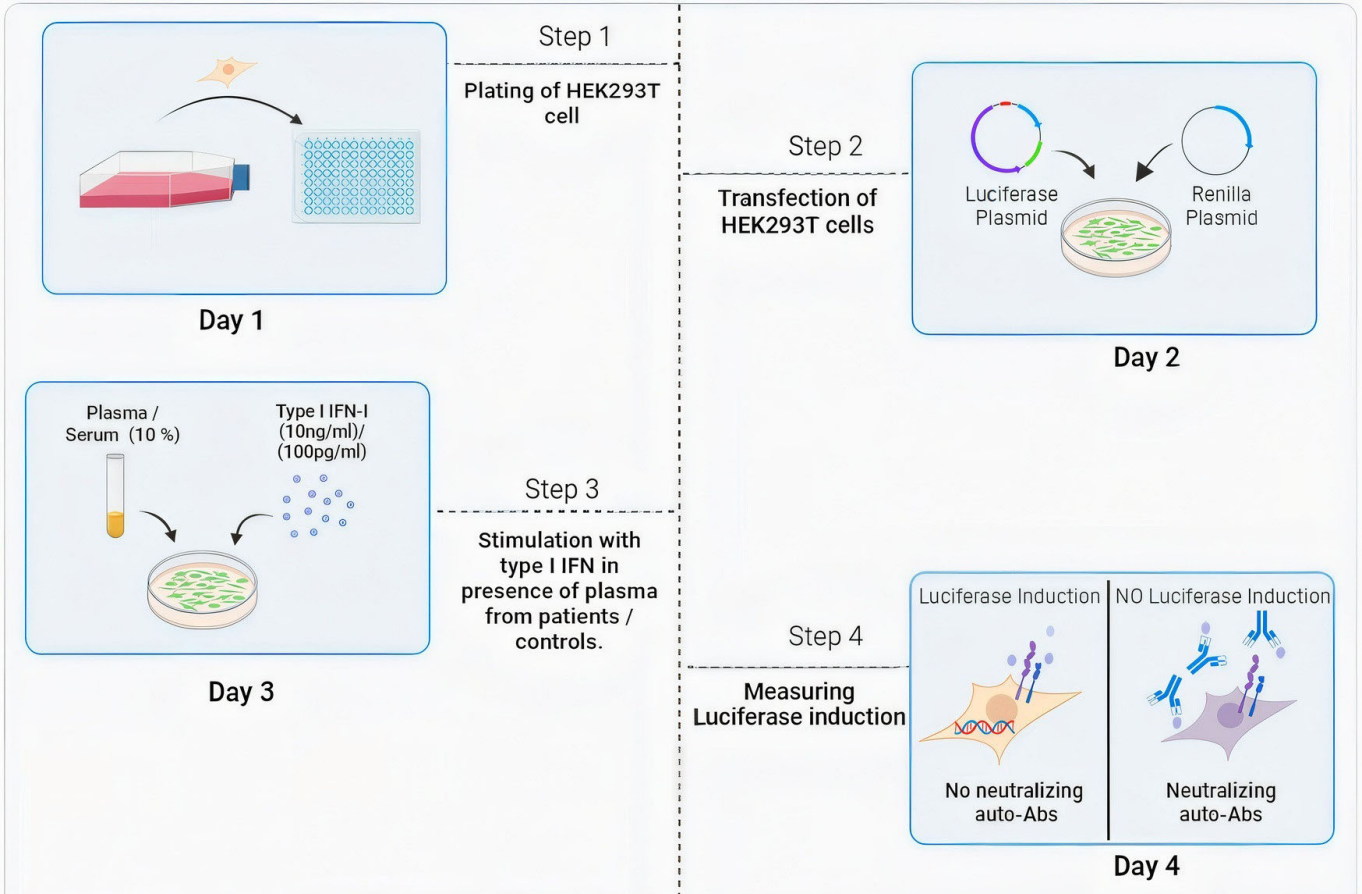
Schematic representation of the neutralization test developed in HEK293T cells using a luciferase system. (Created with BioRender.com).

Cells were cultured in Dulbecco’s modified Eagle medium (DMEM; Thermo Fisher Scientific) supplemented with 2% fetal calf serum and 10% patient plasma, and stimulated with IFN-α2 (Miltenyi Biotec, cat. No. 130-108-984) or IFN-ω (Merck, reference number SRP3061) at concentrations of 10 ng/mL or 100 pg/mL, or recombinant human IFN-β (Peprotech, cat. No. 300-02BC) at 10 ng/mL for 16 hours at 37 °C. Each sample was tested in duplicate for each cytokine and each concentration. Cells were subsequently lysed for 20 minutes at room temperature, and luciferase activity was measured using the Dual-Luciferase Reporter 1000 Assay System (Promega, cat. No. E1980) according to the manufacturer’s protocol. Luminescence intensity was measured using a VICTOR-X Multilabel Plate Reader (Perkin Elmer Life Sciences, USA). Firefly luciferase activity was normalized against Renilla luciferase activity and expressed as a percentage of the median induction level for non-neutralizing control samples. Samples were considered neutralizing if luciferase induction, normalized against Renilla luciferase activity, was <15% of the median value for controls tested on the same day (**Figure 1**).

### 2.6 Statistical analysis

Statistical analyses were performed using IBM SPSS version 21.0 and GraphPad Prism 10. Categorical variables are presented as absolute numbers and percentages; the *Chi-square* or *Fisher’s exact tests* were applied as appropriate. Normality was assessed using the *Shapiro–Wilk* test. Continuous variables are presented as medians with interquartile ranges [Q1–Q3] and were analyzed using *Student’s t-test* or *Mann-Whitney tests*, respectively. A *P*-value <0.05 was considered statistically significant.

### 2.7 Schematic representation

Schematic representations (**Figure 1**) were created using BioRender.com.

## 3 Results

### 3.1 Demographic characteristics

A cohort of 195 patients with critical, severe, moderate or mild COVID-19 was recruited to assess the neutralizing capacity of autoantibodies against low (100 pg/mL) or high (10 ng/ml) concentrations of IFN-α2, IFN-ω, and IFN-β (1ng/mL) in 1:10 diluted plasma. Patient ages ranged from 2 months to 101 years, with a median age of 62 years. Males predominated (126 [64.6%] vs. 69 [35.4%] females), with a male-to-female ratio of 1.8:1. SARS-CoV-2 infection was confirmed in all patients through RT-qPCR. The cohort included 101 (51.8%) critical, 63 (32.3%) severe, 16 (8.2%) moderate and 15 (7.7%) mild COVID-19 cases. None of the patients had received COVID-19 vaccination prior to infection.

The most prevalent comorbidities were hypertension (68 patients, 34.9%), diabetes (54 patients, 27.7%), cardiac disease (18 patients, 9.2%), and obesity (13 patients, 6.7%). Additional comorbidities included dyslipidemia (5 patients, 2.6%), immunosuppression (5 patients, 2.6%), and asthma (5 patients, 2.6%). Consanguinity was documented in 4 patients (2.1%).

### 3.2 Clinical manifestations of patients

**Figure 2:**
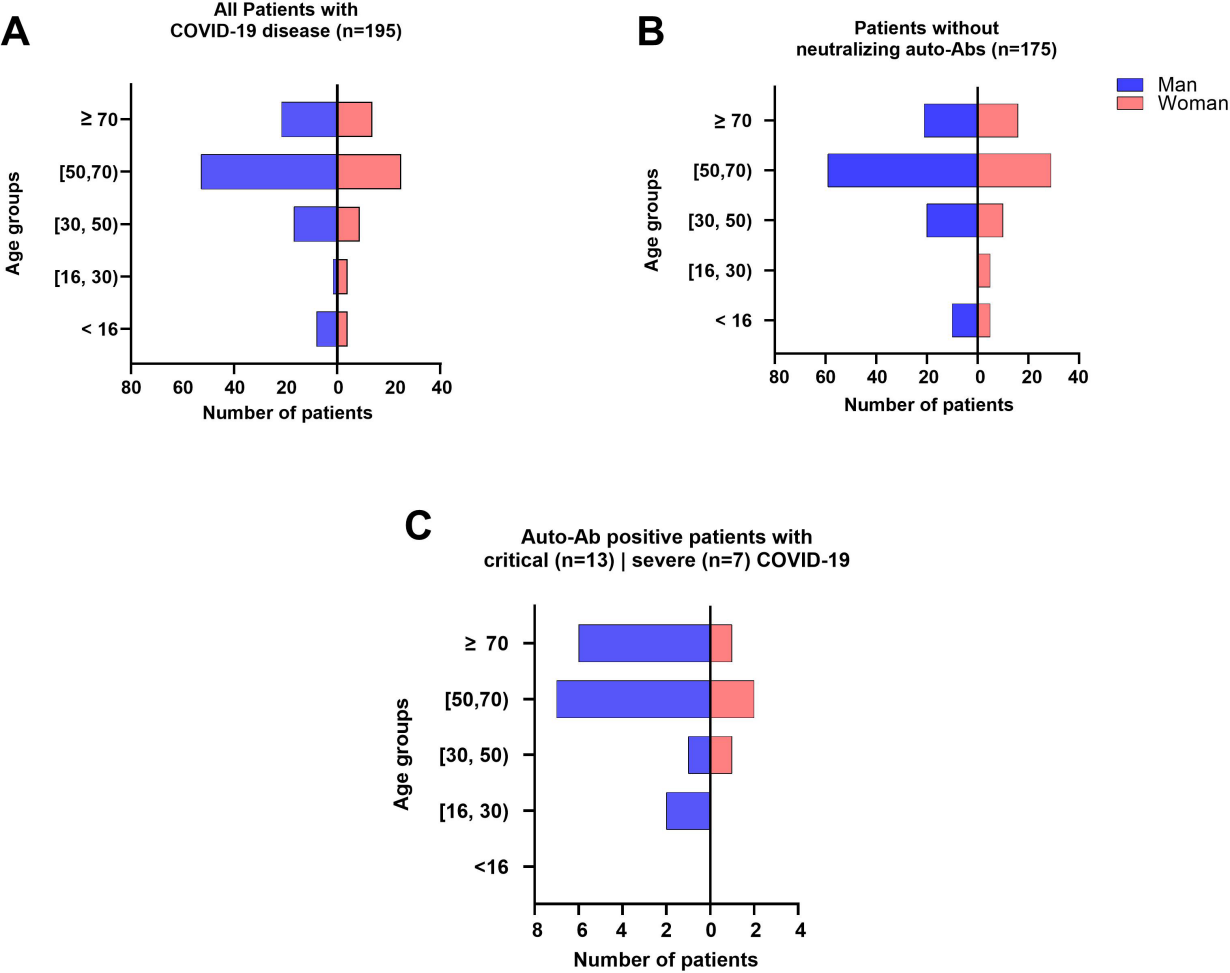
Age and sex distribution in patients with COVID-19 according to the presence of auto-Abs against type I IFNs. **(A)** Age and sex distribution of all included patients (N = 195) with COVID-19. The median age of the COVID19 patients was 62 years (IQR: 49–70 years); 64.6% were males, and 35.4% were females. **(B)** Age and sex distribution of patients without anti-IFN auto-Abs. The median age of the patients was 61 years (IQR: 48–70 years); 62.9% were males, and 37.1% were females. **(C)** Age and sex distribution of individuals with critical, severe positive auto-Abs. The median age of the positive COVID19 patients was 65 years (IQR: 54–77 years); 80% were males, and 20% were females.

Regarding respiratory status, oxygen saturation below 90% was observed in 25/186 patients (13.4%). The median respiratory impairment was 60% [Interquartile range (IQR) 35–75]. Respiratory support requirements varied: 10 (5.1%) patients required face mask ventilation, 35 (17.9%) received nasal cannula oxygen, 8 (4.1%) required invasive mechanical ventilation, and 40 (20.5%) needed non-invasive ventilation. Of note, 16 (8.2%) patients maintained normal spontaneous breathing throughout their hospitalization. High-concentration oxygen therapy was administered to 93 (47.7%) patients during their hospitalization. Comprehensive demographic and clinical characteristics are presented in (**Figure 2**; **Table 1**).

**Table 1:** Demographic, biological characteristics, and clinical features of patients with critical COVID-19.

Variables	Total patients N = 195 (%)	Patients with neutralizing auto-Abs (N = 20)	Patients without neutralizing auto-Abs (N = 175)	P value
Demographic features				
Median age, years old	62.00	65.00	61.00	0.2282
Sex				0.1288
Sex ration	1.8			
Male	126 (64.6%)	16 (80%)	110 (62.9%)	
Female	69 (35.5%)	4 (20%)	65 (37.1)	
Comorbidities				
Hypertension	68 (34.9%)	9 (45%)	59 (34%)	0.662
Diabetes	54 (28%)	6 (30%)	48 (27.4%)	0.436
Cardiac disease	18 (9.2%)	2 (10%)	16 (9%)	0.429
Obesity	13 (6.7%)	1 (5%)	12 (7%)	0.425
Dyslipidemia	5 (5.6%)	1 (5%)	4 (2.3%)	0.432
Immunodepression	5 (2.6%)	0	5 (3%)	0.423
Asthma	5 (2.6%)	1 (5%)	4 (2.3%)	0.432
**Consanguinity**	4 (2%)	0	4 (2%)	
Laboratory markers Median [Q1–Q3]				
C-RP (mg/L) (**N** **=** **148**)	149 [81.30–254]	245.1 [138.3–291.8]	145.5 [77–240]	**0.042**
IL-6 (pg/ml) (**N** **=** **190**)	35.59 [10.65–86.14]	26.21 [6.770–102.8]	35.59 [10.66–85.86]	0.785
Lymphocyte (/mm^3^) (**N** **=** **150**)	795 [525–1180]	710 [510–965]	800 [525–1230]	0.378
Ferritin (ng/mL) (**N** **=** **66**)	604.5 [297.2–1521]	1500 [692.5–1822]	587 (265.9–1425]	0.157
Neutrophil count (/mm³) (**N** **=** **157**)	8770 [5556–12335]	11700 [7630–16394]	8635 (5494–12104]	0.060
Platelet count (x10^3^/mm) (**N** **=** **159**)	291,000[220,000–380,000]	22300[271.3–269250]	103500(288.5– 246500]	0.850
Clinical features				
Critical COVID-19	101 (51.8%)	13 (65%)	88 (50.3%)	
Severe COVID-19	63 (32.3%)	7 (35%)	56 (32%)	
Moderate COVID-19	16 (8.2%)	0	16 (9.1%)	
Mild COVID-19	15 (7.7%)	0	15 (8.6%)	
Oxygen saturation below 90% (n=186)	25 (13.4%)	3 (15%)	22 (13%)	0.077
Median respiratory impairment (**N** **=** **107**) [Q1–Q3]	60% [35; 75]	60% [25; 75]	60 [37–75]	0.505
Face mask ventilation	10 (5.1%)	1(5%)	9 (5.1%)	0.978
Nasal cannula	35 (17.9%)	4 (20%)	31 (18%)	0.800
Invasive mechanical ventilation	8 (4.1%)	1 (5%)	7 (4%)	0.830
Non-invasive ventilation	40 (20.5%)	6 (30%)	34 (19.4%)	0.255
Normal breathing	16 (8.2%)	2 (10%)	14 (8%)	0.757
High-concentration oxygen therapy	93 (47.7%)	12 (60%)	81(46.3%)	0.225

Data are N (%), or median [IQR]/Quantitative values are presented as median [Q1–Q3]; nominal data are indicated as number (percentage). The *P*-value for different variables (age, sex, comorbidities, and clinical features) was analyzed to compare patients with and those without autoantibodies to type I IFNs. Numbers in bold indicate a *P*-value < 0.05.

### 3.3 Laboratory markers of study participants

**Figure 3:**
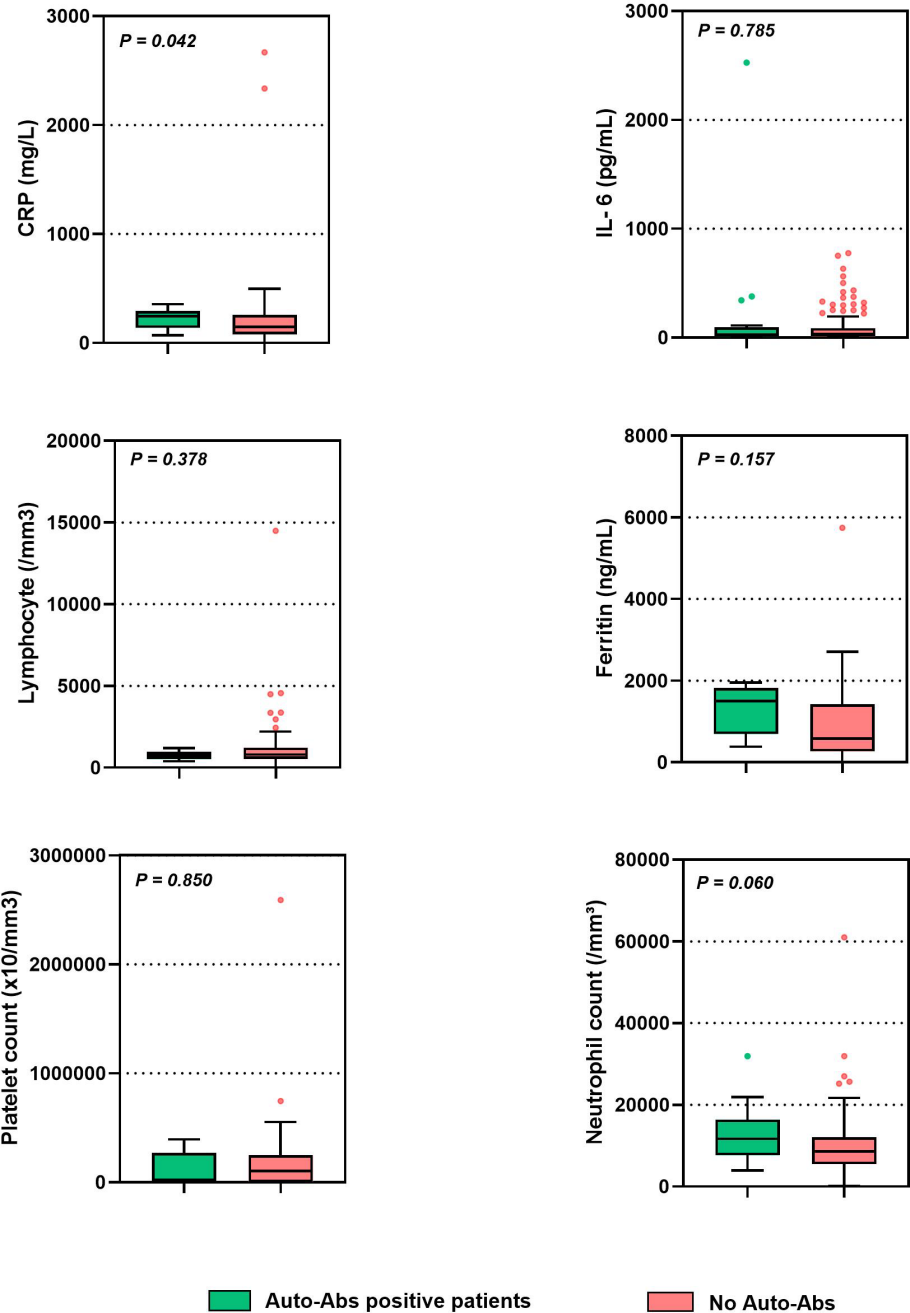
Box chart showing interquartile range (the box), total range (vertical line with spots), and median (horizontal line), comparing biological values in individuals tested positive (green box) or negative (pink box) by the Luciferase-based neutralization assay for auto-Abs neutralizing IFNs. The distribution of CRP, lymphocyte, IL-6, ferritin, platelet count, and neutrophil count is depicted here. The *P*-value of CRP shows a significant difference between the two groups. *p* < 0.05 is considered statistically significant.

Laboratory analysis was performed on available specimens, with results summarized in **Table 1**. Key biomarkers showed the following median values: CRP 149 mg/L (IQR 81.30–254), interleukin-6 35.59 pg/mL (IQR 10.65–86.14), lymphocyte 795 cells/mm³ (IQR 525–1180), ferritin 604.5 ng/mL (IQR 297.2–1521), neutrophil count 8,770 cells/mm³ (IQR 5,556–12,335), and platelet count 291,000 ×10³ cells/mm³ (IQR 220,000–380,000). Patients with neutralizing autoantibodies exhibited significantly elevated CRP levels compared with those without autoantibodies (*P* = 0.042) (**Figure 3**, **Table 1**).

### 3.4 Autoantibodies neutralizing IFN-α2, IFN-ω, and IFN-β in 10.3% of COVID-19 patients

We identified AAN-I-IFNs in 10.3% (20/195) of the patients hospitalized with COVID-19. Among those 20 positive patients, 16 (80%) were male, and 4 (20%) were female, with ages ranging from 19 to 101 years, and the median age was 65 years. AAN-I-IFNs were identified in 13/101 (12.9%) critical patients and 7/63 (11.1%) severe cases. Notably, we did not detect AAN-I-IFNs in patients with moderate or mild COVID-19. The prevalence of auto-Abs in our cohort was consistent with previous findings by Bastard et al. (11, 58) and multiple other studies.

**Figure 4:**
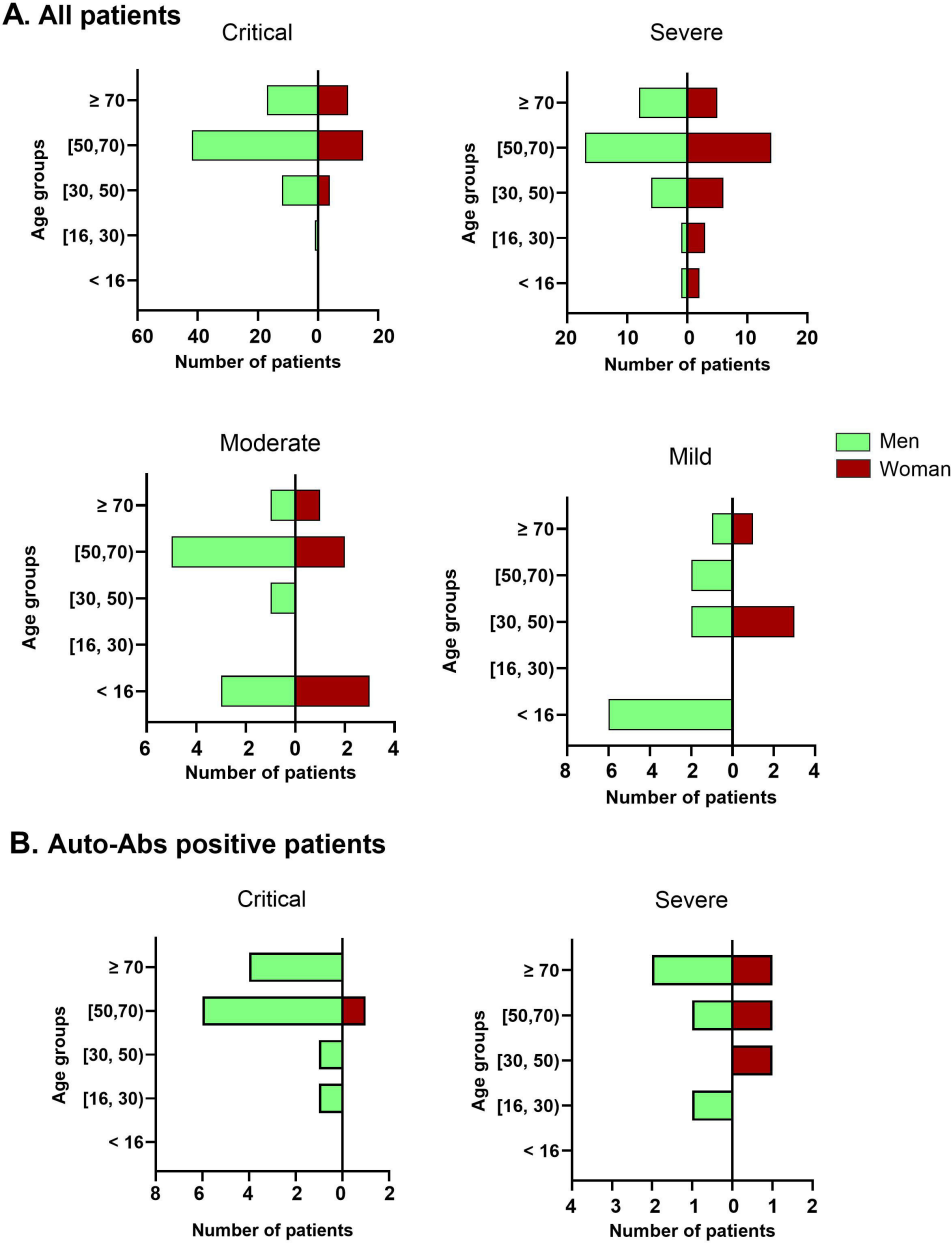
Demographic and phenotypic distributions of the COVID-19 cohort and auto Abs neutralizing type I IFN-α2, IFN-ω, or IFN-β in individuals infected with SARS COV-2 **(A)** Age and sex distribution of the patients with critical (N = 101), severe (n=63), moderate (n=16), and mild (n=15) COVID-19 patients. **(B)** Age and sex distribution of the patients with critical (n=13), severe (n=7) COVID-19 patients with auto-Abs against type I IFNs.

Among the positive cases, none were children (<16 years), 12 were adults aged 19–66 years, and 8 were elderly patients (≥70 years) (**Figure 2C**). No significant age difference was observed between patients with and without auto-Abs (median age 65 vs. 61 years, respectively; (*P* = 0.2282) (**Figures 2B, C**; **Table 1**). Regarding sex distribution within disease severity groups, 12 of the 13 of AAN-I-IFN-positive patients with critical COVID-19 were male, whereas only one was female (**Figure 4B**). In contrast, AAN-I-IFN-positive patients with severe COVID-19 showed a more balanced sex distribution, including four males (aged 19–81 years) and three females (aged 39–77 years) (**Figure 4B**). No significant sex difference was observed between auto-Abs-positive and auto-Abs-negative groups (*P* = 0.1467).

**Figure 5:**
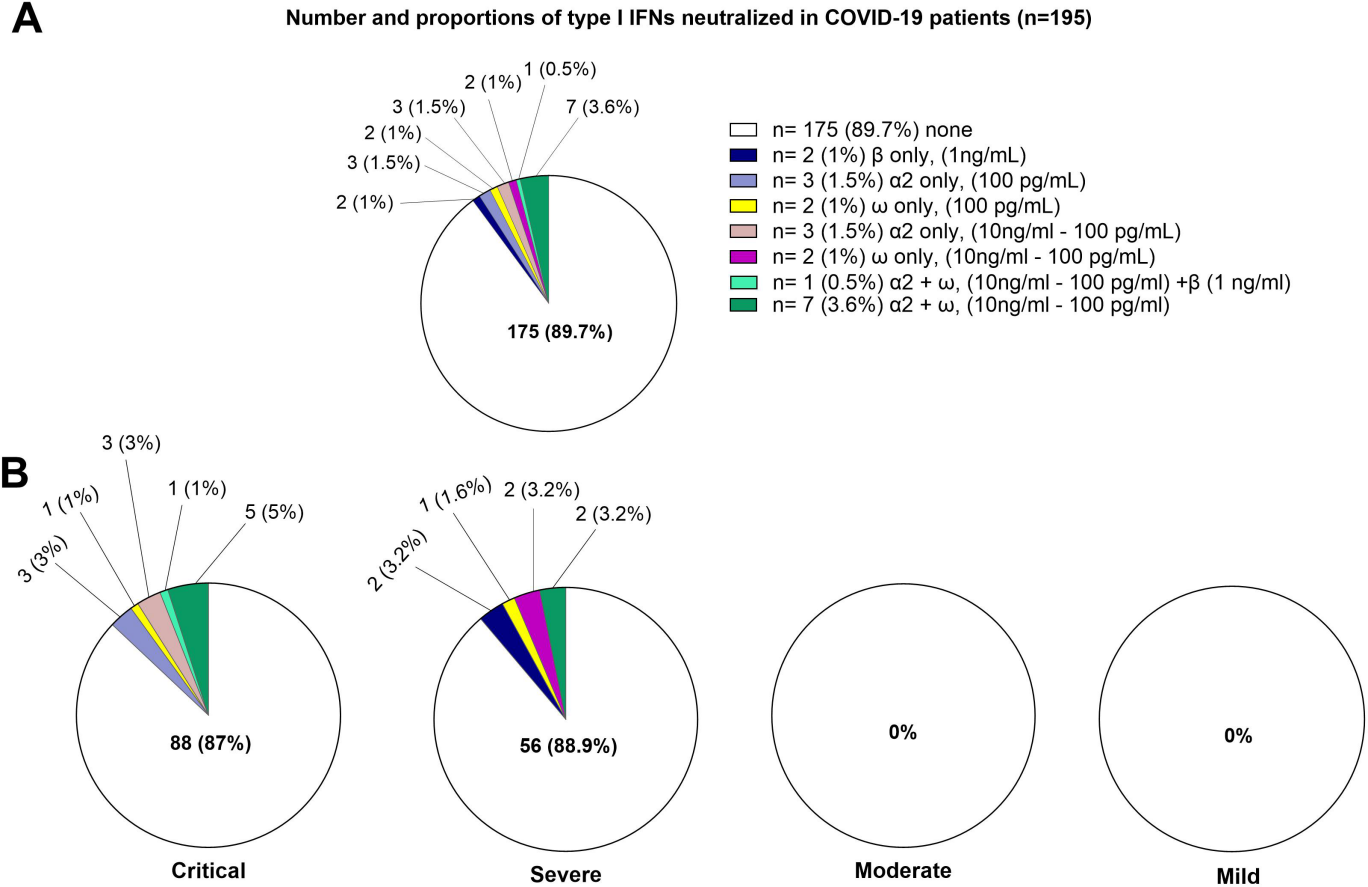
Number (n) and proportion (%) of type I IFNs neutralized in COVID-19 patients according to the nature and combination of auto-Abs. **(A)** Proportion of type I IFNs neutralized in 195, which was included in our study. **(B)** Proportion of type I IFNs neutralized in critical (N = 13/101), severe (N = 7/63), moderate (N = 0/16), or mild (N = 0/15) in individuals with COVID-19.

**Figure 6:**
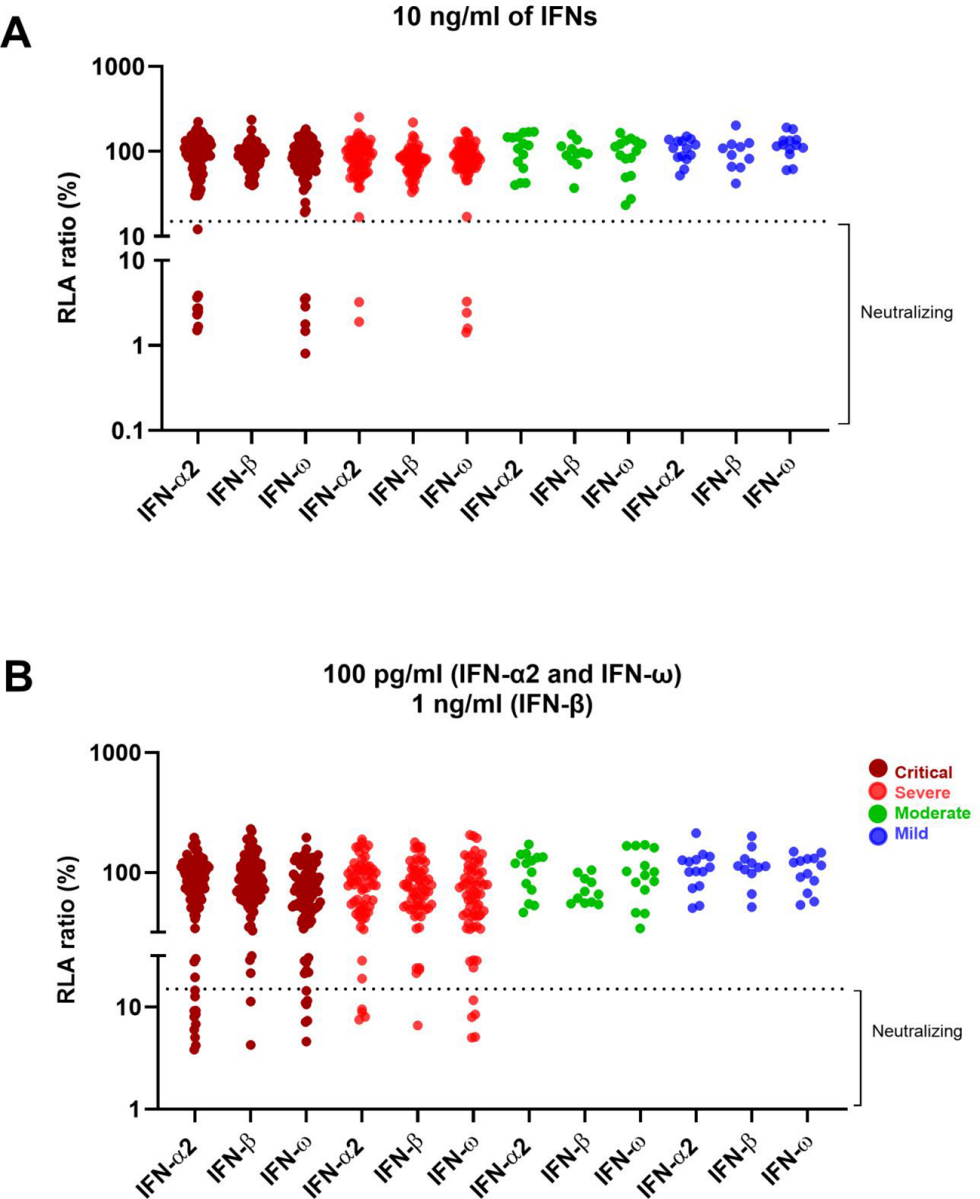
Auto-Abs neutralizing IFN-α2 and/or IFN-ω and/or IFN-β in patients with critical, severe, moderate or mild COVID-19. **(A)** Luciferase-based neutralization assay to detect auto-Abs neutralizing 10 ng/mL IFN-α2, IFN-ω, or IFN-β (top panel) or **(B)** 100 pg/mL IFN-α2 or IFN-ω, or 1 ng/mL IFN-β (bottom panel). Plasma samples from patients with critical (garnet red), severe (red), moderate (green), or mild (blue) COVID-19 were diluted 1/10 in all tests. HEK293T cells were transfected with a plasmid containing the firefly luciferase gene under the control of an IFN-sensitive response element (ISRE)-containing promoter and a plasmid containing the Renilla luciferase gene. The cells were then treated with type I IFNs, and relative luciferase activity (RLA) was calculated by normalizing firefly *Renilla* luciferase activity. An RLA <15% of the value for the mock treatment was considered to correspond to neutralizing activity. Each sample was tested at least twice.

**Figure 7:**
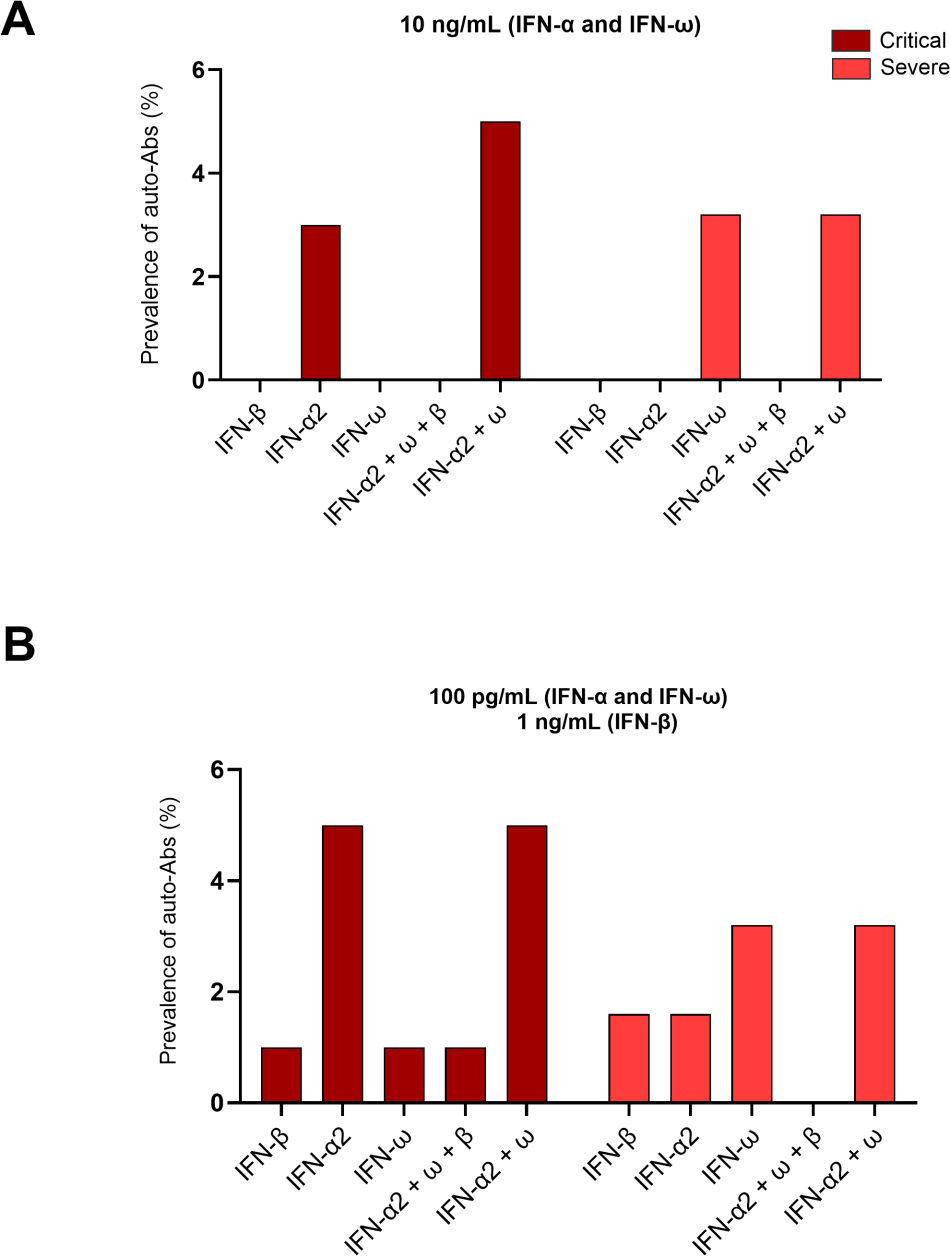
Proportion of individuals with auto-Abs neutralizing type I IFNs. **(A)** At higher (10 ng/mL) (up), or **(B)** lower concentration (100 pg/mL)/1ng/mL (bottom) in the critical (n=101), severe (n=63), of COVID-19 patients. COVID-19 individuals were screened for auto-antibodies neutralizing type I IFNs using a luciferase-based neutralization assay. None of the patients with moderate or mild groups carry auto-antibodies against type I IFNs. Critical cases are shown in garnet red, severe in red.

None of the mild and moderate cases had detectable AAN-I-IFN neutralizing high (10 ng/mL) or low concentrations (100 pg/mL)/1ng/mL of the three type I IFNs tested (**Figures 5B**, **6A, B**, **7**).

Among the 20 AAN-I-IFN-positive patients, two of the 195 (1.03%) patients had auto-Abs exclusively neutralizing IFN-β at 1 ng/mL, 7 (3.6%) neutralized low and high concentrations of IFN-α2 and IFN-ω, and 2 (1%) neutralized high and low concentrations of IFN-ω only. For IFN-α2 only, 3 (1.5%) patients neutralized 10ng/mL and 100 pg/mL, while 2 (1%) neutralized low concentrations of IFN-ω only, and 3 (1.5%) neutralized low concentrations of IFN-α2 only. Remarkably, 1 (0.5%) patient neutralized all three interferons tested (IFN-α2, IFN-ω, and IFN-β) at 10 ng/mL (**Table 2**; **Figure 5**).

**Table 2:** Proportion and number of patients with auto-abs neutralizing IFN-α2 and/or IFN-ω and/or IFN-β in plasma (1:10).

Type and concentration of type I IFNs in the plasma	Number (%) of patients with neutralizing activity
IFN-β auto-Abs only (1ng/mL)	2 (1%)
IFN-α2 and IFN-ω (10 ng/mL- 100pg/mL)	7 (3.6%)
IFN-ω auto-Abs only (10 ng/mL- 100pg/mL)	2 (1%)
IFN-α2 auto-Abs (10 ng/mL- 100pg/mL)	3 (1.5%)
IFN-ω auto-Abs only (100pg/mL)	2 (1%)
IFN- α2 auto-Abs only	3 (1.5%)
IFN-α2 and IFN-ω (10 ng/mL - 100pg/mL) and IFN-β auto-Abs (1 ng/mL)	1 (0.5%)

Comorbidity analysis among the 20 AAN-I-IFN-positive patients revealed hypertension in 9 out of 20 (45%) patients, diabetes in 6/20 (30%), cardiac disease in 2/20 (10%), and obesity in 1 (5%) patient. Additional conditions included dyslipidemia in 1 (5%) and asthma in 2 (10%) patients. Rare comorbidities observed included ischemic stroke, end-stage renal disease, ischemic heart disease, glaucoma, and gout in four critically ill patients (P2, P15, P8, P16), while irritable bowel syndrome, cataract, and Duchenne muscular dystrophy were documented in patients with varying disease severity (patients P13, P19) (**Table 3**). Importantly, no statistically significant differences in comorbidity profiles were observed between patients with and without autoantibodies (**Table 1**).

**Table 3:** Demographic and clinical characteristics of 20 (10.3%) COVID-19 patients with auto-Abs neutralizing type I IFN included in this study.

Patient no.	Auto-Ab positivity						Sex	Age (yr)	COVID-19 severity	Clinical history
	IFN-α (10ng/mL)	IFN- α (100pg/mL)	IFN-ω (10ng/mL)	IFN-ω (100pg/mL)	IFN-β (1ng/mL)	IFN-β (10ng/mL)				
P1	**+**	**+**	**+**	**+**	**-**	**-**	M	101	Critical, ICU, High-concentration mask	Diabetes, Cardiopathy
P2	**+**	**+**	**+**	**+**	**+**	**-**	M	81	Critical, ICU, High-concentration mask,	Hypertension, Ischemic Stroke, ESRD
P3	**+**	**+**	**+**	**+**	**-**	**-**	M	77	Critical, ICU, invasive mechanical ventilation, non-invasive ventilation, High-concentration mask	hypertension, Dyslipidemia
P4	**-**	**+**	**-**	**-**	**-**	**-**	M	70	Critical, ICU, Face mask ventilation,	Diabetes, hypertension
P5	**+**	**+**	**-**	**-**	**-**	**-**	M	66	Critical, ICU, 15L O2, non invasive ventilation, High-concentration mask	
P6	**+**	**+**	**-**	**-**	**-**	**-**	M	64	Critical, ICU, 15L O2, High-concentration mask	Diabetes, hypertension
P7	**+**	**+**	**+**	**+**	**-**	NA	F	62	Critical, ICU, High-concentration mask	
P8	**-**	**+**	**-**	**-**	**-**	NA	M	54	Critical, ICU, High-concentration mask	Glaucoma
P9	**-**	**+**	**-**	**-**	**-**	**-**	M	54	Critical, ICU, High-concentration mask, non invasive ventilation	hypertension
P10	**+**	**+**	**+**	**+**	**-**	**-**	M	58	Critical, ICU, non invasive ventilation, High-concentration mask	
P11	**-**	**-**	**-**	**+**	**-**	**-**	M	42	Critical, ICU, High-concentration mask	
P12	**+**	**+**	**+**	**+**	**-**	**-**	M	81	Severe, ICU, non invasive ventilation,	Diabetes, hypertension
P13	**-**	**-**	**-**	**-**	**+**	**-**	M	80	Severe, ICU, nasal cannula	IBS, Cataract
P14	**+**	**+**	**+**	**+**	**-**	**-**	F	77	Severe, ICU, nasal cannula	hypertension, Asthma
P15	**+**	**+**	**-**	**-**	**-**	**-**	M	72	Severe, ICU, High-concentration mask	IHD, hypertension
P16	**-**	**-**	**-**	**-**	**+**	**-**	M	66	Severe, ICU, non invasive ventilation, High-concentration mask	Gout, hypertension, Diabetes
P17	**-**	**-**	**+**	**+**	**-**	**-**	F	58	Severe, ICU, nasal cannula	Obesity
P18	**-**	**-**	**-**	**+**	**-**	NA	F	39	Severe, nasal cannula	
P19	**-**	**-**	**+**	**+**	**-**	**-**	M	19	Severe, ICU, High-concentration mask,	DMD
P20	**+**	**+**	**+**	**+**	**-**	**-**	M	24	Critical, ICU, nasal cannula	Diabetes,

+, positive; -, negative; F, female; M, male; ICU, intensive care unit; ESRD, End-Stage Renal Disease; IHD, Ischemic heart disease; IBS, Irritable Bowel Syndrome; DMD, Duchenne Muscular Dystrophy; NA, data not available.

### 3.5 Demographic characteristics of Moroccan AAN-I-IFN-positive patients

Among the 20 AAN-I-IFN-positive patients, the median age was 65 years (IQR 54–77), ranging from 19 to 101 years, and 16 (80%) were male, with no pediatric patients aged under 16 years. Auto-Abs against IFN-α2 and/or IFN-ω were the most commonly identified. Specifically, 7/20 (35%) individuals neutralized both high and low concentrations of IFN-α2 and IFN-ω (median age 68 years, range 24–101 years), with males (n=5) showing a higher prevalence than females (n=2). Three individuals (aged 64–72 years) carried auto-Abs neutralizing both high and low concentrations of IFN-α2 only, all of whom were male. Two patients (aged 19 and 58 years) neutralized IFN-ω at both 10 ng/mL and 100 pg/mL. In the three patients with auto-Abs neutralizing IFN- α2 (100 pg/mL), the age ranged from 54 to 70 years. Two individuals aged 39–42 years, and carried auto-Abs neutralizing low concentrations of IFN- ω only. Auto-Abs neutralizing IFN-β (1 ng/mL) alone were identified in two male patients (66–80 years). At more physiological concentrations, specimens from only one patient (81 years) contained auto-Abs neutralizing IFN-α2 and IFN-ω and 1 ng/mL IFN-β. The distribution of AAN-I-IFNs across subtypes and concentrations is shown in **Table 3**. Although younger patients may carry AAN-I-IFNs (as young as 19 years in the present study), their frequency increases substantially after the age of 70.

## 4 Discussion

We found that 10.3% of patients hospitalized with severe or critical COVID-19 in a Moroccan cohort had auto-Abs neutralizing IFN-α2, and/or IFN-ω, and/or IFN-β, whereas none were observed in patients with mild or moderate disease. We observed no significant differences in terms of age, sex, or comorbidities between patients with or without auto-Abs. The proportion of women with detectable auto-Abs in our study was 20%, consistent with previous findings. Notably, we found no significant difference between critical patients with and without auto-Abs regarding oxygen respiratory support requirements. However, during hospitalization, there was a trend toward increased oxygen therapy requirements in patients with AAN-I-IFN. These findings underscore the importance of close monitoring and intensive respiratory support for critical COVID-19 patients harboring AAN-I-IFN. The present study focused on clinical manifestations in AAN-I-IFN-positive patients; however, multiple studies (20, 23, 40, 45) have demonstrated a significant association between AAN-I-IFN and mortality in critical COVID-19.

In the present study, no significant differences were observed in laboratory values, including IL-6, lymphocyte, ferritin, neutrophil count, or platelet count (**Table 1**). Nevertheless, there was a significant difference in CRP level (*P =* 0.042) between patients with and without AAN-I-IFNs. Solanich et al. (45) found auto-Abs against type I IFN-α2 and IFN-ω by ELISA in approximately one-fifth of 275 ICU-admitted COVID-19 patients in Barcelona. They reported statistically significant neutrophilia, leukocytosis, and thrombocytosis in COVID-19 patients with auto-Abs against type I IFNs (45). Similarly, Troya et al. showed significantly elevated CRP levels, as well as lower lymphocyte values, in patients with type I IFN-alpha- and omega-neutralizing auto-Abs (n=5) among 47 COVID-19 patients. Of note, Staudacher et al. (59) described a correlation between AAN-I-IFN and low CD169/SIGLEC1 expression on monocytes in patients with acute SARS-CoV-2 infection. In their study of 808 German emergency department patients, AAN-I-IFN were associated with worse clinical outcomes and increased mortality. Patients presenting to the emergency department with high CRP (>50 mg/L) and low CD169/SIGLEC1 expression had a 70% prediction rate of harboring AAN-I-IFN, suggesting this dual-biomarker combination as a rapid flow cytometry-based screening approach for AAN-I-IFNs in COVID-19. These differences in laboratory values between the two groups may help identify individuals at greater risk of developing critical COVID-19 and consequently, facilitate timely intervention with appropriate therapeutic strategies.

Previous studies have detected few or no AAN-IFN-β (11, 20, 40, 60). Interestingly, among patients in our cohort, auto-Abs against IFN-β (1 ng/mL) alone or in combination with IFN-α2 and IFN-ω were detected in three patients (15%) with critical (n=2) or severe (n=1) COVID-19. Based on Bastard et al. (11, 52), AAN-IFN-β were identified in 1.3% of critical COVID-19 patients. Although rare, these auto-Abs are associated with critical COVID-19 and may independently compromise antiviral immunity. The higher proportion of anti-IFN-β auto-Abs in our study likely reflects the large number of critically ill patients, which may partially explain the differences from prior reports. As previously reported, of 101 critical patients with AAN-I-IFN, only two had detectable auto-Abs neutralizing IFN-β with IFN-α2 and/or IFN-ω (11). Accordingly, a total of 1773 critically ill COVID-19 patients were assessed for the presence of auto-Abs against IFN-β, and their presence was demonstrated in 1.3% (23/1773). Conversely, they were found in only two of 1044 (0.18%) controls tested but in none of the samples from 187 severe patients (52). In this regard, Troya et al. (40) investigated the prevalence of AAN-I-IFN in 47 hospitalized patients with severe COVID-19 treated with IFN beta-1b, and they observed undetectable auto-Abs against IFN-β. A study by Arrestier et al. (16) encompassing several centers in France reported a higher prevalence of women admitted to intensive care units with positive auto-Abs of 21.9% (21/96), and auto-Abs against IFN-β (33.3%) were more common in young women exhibiting an autoimmune background. Further investigations in larger, independent cohorts are warranted to determine whether this finding is specific to our cohort or reflects a broader pattern, particularly in Moroccan patients, and to clarify the potential therapeutic implications of IFN-β involvement in COVID-19 pathophysiology.

We did not screen for the presence of AAN-I-IFN in healthy individuals’ samples, and only hospitalized patients with proven SARS-CoV-2 infections were included in our research. However, the pioneer study of Bastard et al. (11) described and already showed in the plasma and serum samples of 1227 healthy individuals who had not been exposed to SARS-CoV-2 infection that auto-Abs were found in only 4/1227 (0.33%) of healthy controls. Indeed, they have been found in 0% of 663 patients with asymptomatic or mild disease (11). In line with this, multiple studies demonstrated that neutralizing auto-Abs against type I IFNs are detected in a minor proportion of healthy controls, and never in mild or asymptomatic COVID-19. In a Peruvian cohort of hospitalized patients with severe/critical COVID-19, the presence of anti-IFN-α auto-Abs was observed in 26/54 (48.2%) patients with severe/critical COVID-19 (31). In contrast, they were not detected in asymptomatic or mild COVID-19, with auto-Abs detected in 3.1% of healthy groups (31). Likewise, a Spanish study reported in hospitalized adult patients asymptomatic (0/118) controls with type I IFN-α and IFN-ω neutralizing auto-Abs (40). In a French study of critically ill COVID-19 patients admitted to intensive care units similarly revealed that undetectable anti-IFN-α auto-Abs were present in patients with milder stages of the disease (20). Several cohort series replicated these findings. Overall, as previously reported by published studies, AAN-I-IFN were detected in a significant proportion (10-20%) of hospitalized patients with severe/critical COVID-19. However, they were less detectable in uninfected healthy controls (0.33%) or even absent in the asymptomatic/mild (0%) cases. This suggests that the high titer of AAN-I-IFN in severe/critical cases was associated with disease severity and pathogenesis, as well as strongly representing a risk marker for adverse outcomes. Of note, the nature of our inclusion criteria limits the generalizability of our findings among North African individuals; by focusing only on hospitalized patients, as well as the lack of uninfected healthy controls and small samples remains a significant limitation of the current study, and underscores the need for further investigations with the inclusion of much larger cohorts with uninfected individuals to better explain and evaluate the observed prevalence of AAN-IFN-β in future clinical studies and to select the more suitable therapies.

Current identification of AAN-I-IFN has important clinical implications. First, testing for these auto-Abs is strongly recommended for the elderly and patients with severe autoimmune and inflammatory diseases. Second, plasma exchange therapy (plasmapheresis) should be considered in individuals with severe viral disease to eliminate auto-Abs from circulation. The association of antiviral drugs and plasmapheresis is important in patients with severe respiratory disease. Third, treatment with type I IFNs has been explored as a therapeutic approach to reduce pathological inflammation in respiratory diseases; however, this strategy may carry a risk of inducing anti-IFN-β auto-Abs in vulnerable patients. However, early treatment with type I IFNs can be effective and play a crucial role in reducing clinical symptoms. Fourth, vaccination should be prioritized for patients with positive AAN-I-IFN, although live attenuated vaccines should be avoided. Fifth, accessible diagnostic tests for neutralizing auto-Abs against type I IFNs are valuable for screening of patients with a history of severe viral infection. Finally, inflammatory markers such as CRP represent emerging biomarkers with potential clinical relevance in patients with AAN-I-IFN; future studies should further characterize the roles of additional inflammatory mediators (e.g., interleukin-1β, chemokines, insulin-like growth factor, tumor necrosis factor-α) to improve prognostic accuracy.

## 5 Concluding remarks

Type I IFNs play a pivotal role in the defense against viruses and coordinate downstream immune response by limiting viral replication. The confirmed titer of AAN-I-IFNs among multiple populations and with a higher prevalence (10-20%) in severe/critical illness compared to asymptomatic/mild cases provides evidence that impaired type I IFN or response may disrupt these protective mechanisms, contributing to uncontrolled viral replication. In summary, our findings are consistent with previous studies and provide robust real-world evidence supporting the critical role of AAN-I-IFN in 10.3% of Moroccan patients with life-threatening COVID-19. We observed a higher-than-expected proportion of patients with auto-Abs neutralizing IFN-β (alone or in combination with IFN-α2/IFN-ω), particularly in critical and severe disease, compared to the 1.3% and 0.9% rates reported in prior studies of critical and deceased patients. In this sense, our study raises many additional questions that deserve to be addressed in the years ahead. These questions can only be addressed by serum collection from multiple geographic regions of Morocco, creating sizable cohorts and much larger numbers of participants from the North African population, and prospectively enrolling individuals with new-onset viral disease, such as respiratory conditions, to improve the course of the disease in hospitalized patients with viral disease.

## Data Availability

The raw data supporting the conclusions of this article will be made available by the corresponding authors upon request, without undue reservation.

## Glossary

AAN-I-IFNs: Autoantibodies neutralizing type I interferons
CoVID-19: Coronavirus disease 2019
SARS-CoV-2: Severe Acute Respiratory Syndrome Coronavirus 2
Auto-Abs: Autoantibodies
WHO: World Health Organization
IFNs: Interferons
IFN-α: Interferon-α
IFN-β: Interferon-β
IFN-ε: Interferon-ε
IFN-κ: Interferon-κ
IFN-ω: Interferon-ω
VZV: Varicella zoster virus
MERS: Middle east respiratory syndrome
APS-1: Autoimmune polyendocrine syndrome type 1
WNV: West Nile virus
TBE: Tick-borne encephalitis
POW: Powassan
USU: Usutu
FVH: Fulminant viral hepatitis
HIV-1: Human immunodeficiency virus
TLR3: Toll-like receptor 3
IFNAR1: Interferon alpha and beta receptor subunit 1
TLR7: Toll-like receptor7
ICU: Intensive care unit
RT-qPCR: Reverse transcription-quantitative polymerase chain reaction
RNA: Viral Ribonucleic acid
MAScIR: Moroccan Foundation for Ad-vanced Science Innovation and Research
RdRp: RNA-dependent RNA polymerase
S: Spike
dNTP: deoxynucleotide triphosphates
PCR: Polymerase chain reaction
Ct: Cycle threshold
N: Nucleocapsid
E: Envelope
SpO_2_: Peripheral capillary oxygen saturation
IL-6: Interleukin-6
EDTA: Ethylenediamine Tetra-acetic Acid
CRP: C-Reactive Protein
LRA: Luciferase Reporter Assays
HEK293T: Human embryonic kidney cells
ISRE: Interferon-stimulated response element
IQR: Interquartile range
Q1: quartile 1
Q3: quartile 3
DMEM: Dulbecco's modified Eagle medium
IBM: International business machines
SPSS: Statistcal package for social sciences
CD169: Cluster of differentiation 169
SIGLEC1: Sialic acid binding immunoglobulin-like lectin 1
